# Genetic liability to mental disorders in relation to the risk of hypertension

**DOI:** 10.3389/fcvm.2023.1087251

**Published:** 2023-02-27

**Authors:** Ning Huangfu, Yunlong Lu, Hongchuang Ma, Ziwei Hu, Hanbin Cui, Fangkun Yang

**Affiliations:** ^1^Department of Cardiology, Ningbo First Hospital, School of Medicine, Ningbo University, Ningbo, China; ^2^Key Laboratory of Precision Medicine for Atherosclerotic Diseases of Zhejiang Province, Ningbo, China; ^3^Cardiovascular Disease Clinical Medical Research Center of Ningbo, Ningbo, China; ^4^Department of Cardiology, The First Affiliated Hospital, School of Medicine, Zhejiang University, Hangzhou, China

**Keywords:** depression, neuroticism, hypertension, mental health, causal association

## Abstract

**Background:**

Observational studies have indicated that psychosocial factors contribute to hypertension; however, the causality of these associations remains unclear due to reverse causality and confounders. We aim to assess the causal associations of mental health disorders with hypertension.

**Methods:**

Instrumental variables of anxiety disorder, attention deficit/hyperactivity disorder, autism spectrum disorder, depression, obsessive–compulsive disorder, post-traumatic stress disorder, schizophrenia, and subjective well-being measure were obtained from the corresponding largest genome-wide association studies. Summary statistics for the association of essential hypertension were obtained from the FinnGen Study (42,857 cases and 162,837 controls) and UK Biobank cohort (54,358 cases and 408,652 controls). The multiplicative random-effects inverse-variance weighted method was utilized as the primary analysis and three other statistical methods were conducted in the supplementary analyses. The results were combined using the fixed-effects method.

**Results:**

In the pooled analyses, genetic liability to depression was associated with higher risk of hypertension (odds ratio [OR], 1.25; 95% confidence interval [CI], 1.17–1.35; *p* < 0.001). Besides, a suggestive association was found between genetically predicted higher weighted neuroticism sum-score and increased risk of hypertension (OR, 1.16; 95% CI, 1.02–1.33; *p* < 0.05). No associations were found for other mental health disorders. Sensitivity analyses revealed consistent evidence as the main results.

**Conclusion:**

We provide consistent evidence for the causal effect of genetic liability to depression on hypertension, which highlights the importance of blood pressure measurement and monitoring in patients with depression.

## Introduction

Approximately, 1.2 billion people worldwide suffer from hypertension (1), which is an important risk factor for stroke, ischemic heart disease, and kidney disease. Although many observational studies have indicated that psychosocial factors may contribute to hypertension (2–4), these findings may be subjected to incomplete adjustment for confounding factors and divergent definitions of mental health, which therefore hinders the causal inference in these associations. Furthermore, as patients with hypertension often have mental health morbidities (5), it appeared to be difficult to diminish reversal causality in observational studies.

Mendelian randomization (MR) analysis sets the basis on Mendel’s second law of inheritance, which can strengthen the casual inference in an association between an exposure and an outcome by using genetic variants as instrumental variables (6). Resembling randomized controlled trials, MR randomizes participants into groups since genetic variants are randomly assigned to offspring and perpetually maintains stable and MR analysis can thus reduce confounders and reverse causation bias.

In recent years, a variety of genome-wide association studies (GWAS) have been published for identification of genetic risk loci for psychosocial factors (7–15), which provides a promising basis to evaluate the contribution of mental health to hypertension from a genetic perspective. Here, we resorted to the two-sample MR design to investigate the causal associations between genetic liability to mental health disorders, including anxiety disorder, attention deficit/hyperactivity disorder, autism spectrum disorder, depression, obsessive–compulsive disorder, post-traumatic stress disorder, schizophrenia, and subjective well-being measure with hypertension risk.

## Methods

### Study design

**Figure 1:**
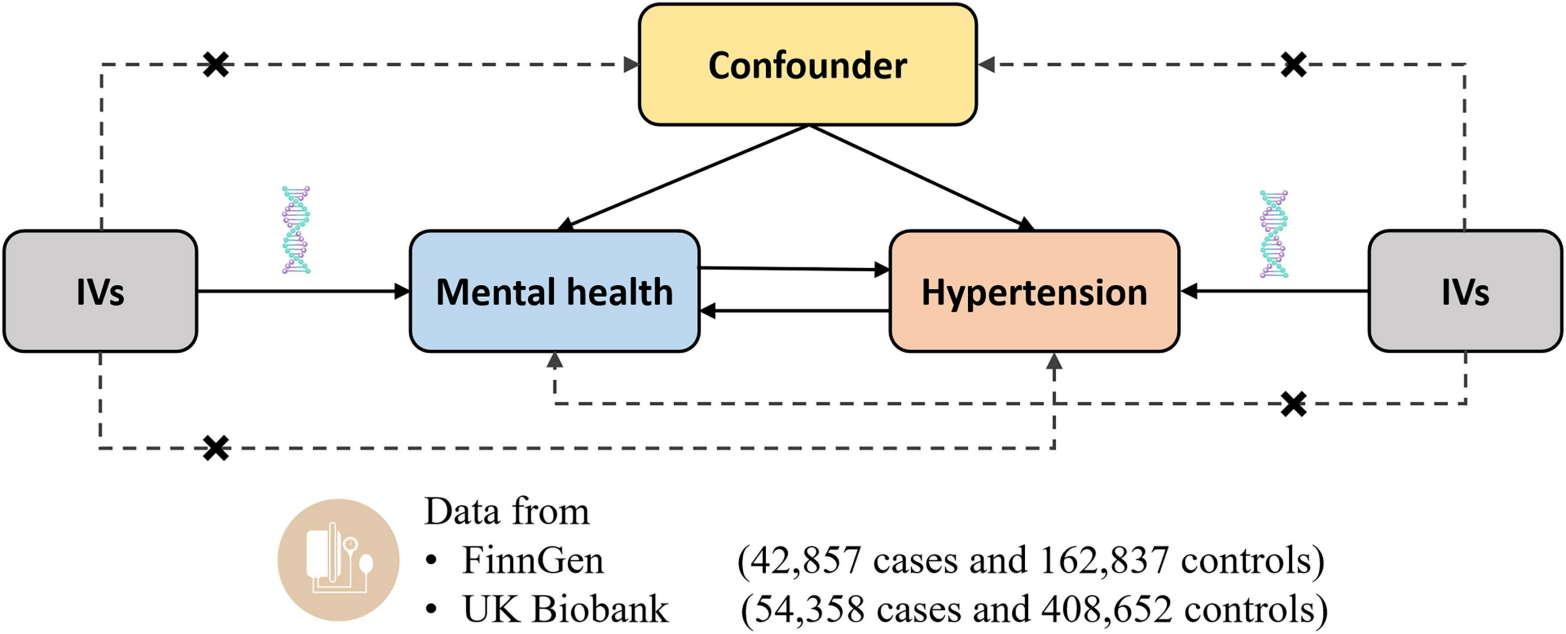
Design of the current two-sample Mendelian randomization study. Three core assumptions were as follows: (*α*) Relevance assumption; (*β*) Independence assumption; (*γ*) Exclusion restriction. IVs, instrumental variables.

The current study was a two-sample MR study to assess the causal associations between mental health and the risk of hypertension using genetic data obtained from the publicly available datasets (Figure 1). Instrumental variables for the mental health disorders should satisfy the following three key assumptions: (I) Relevance assumption, i.e., the genetic variants should be strongly associated with mental health, (II) Independence assumption, i.e., the genetic variants should be independent of potential confounders, and (III) Exclusion restriction, i.e., the genetic variants should only be associated with the risk of hypertension only through the change of mental health. Each study included in this analysis was approved by the corresponding ethics committee.

### Genetic instrument selection

For the mental health disorders (regarded as exposures in the current study), single nucleotide polymorphisms (SNPs) associated at the genome-wide significance threshold of *p* < 5 × 10^−8^ were selected. The SNPs were pruned for linkage disequilibrium tests at *r*^2^ < 0.1, and the SNP with the lowest *p* value were retained as the instrumental variable. The estimates were queried in the outcome (hypertension) GWAS by matching for SNPs of exposure-related instrumental variables. If no corresponding match was found, a proxy at linkage disequilibrium (*r*^2^ > 0.8) was used to approximate it through an online tool named SNiPa (available at http://snipa.helmholtz-muenchen.de/snipa3/).

**Figure 2:**
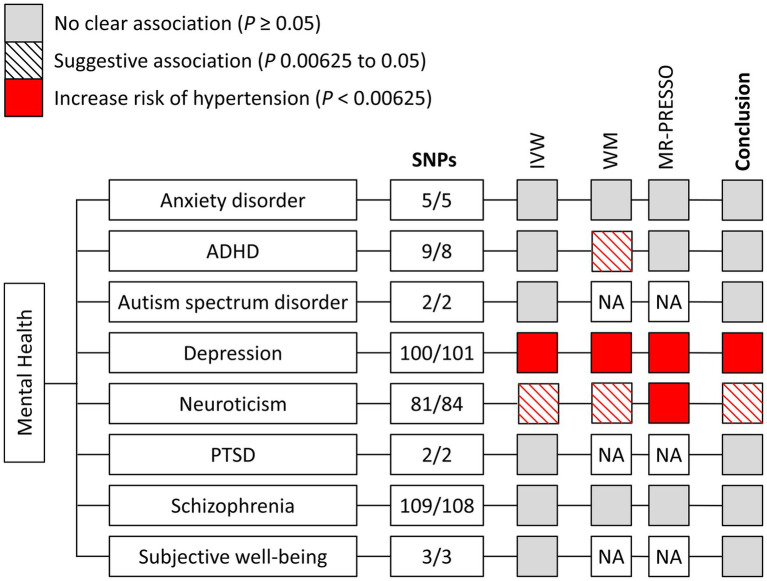
Mendelian randomization associations of genetically determined mental health with hypertension using different statistical models. ADHA indicates attention deficit/hyperactivity disorder; IVW, inverse-variance weighted; MR-PRESSO, Mendelian randomization-pleiotropy residual sum and outlier; NA, not available; PTSD, post-traumatic stress disorder; SNP, single nucleotide polymorphism; and WM, weighted median.

Following the approach described above, we identified 5/5 SNPs (FinnGen/UK Biobank) for anxiety disorder (7), 9/8 SNPs for attention deficit/hyperactivity disorder (ADHD) (8), 2/3 SNPs for autism spectrum disorder (9), 100/101 SNPs for depression (10), 81/84 SNPs for neuroticism (11), no SNP for obsessive–compulsive disorder (12), 2/2 SNPs for post-traumatic stress disorder (PTSD) (13), 109/108 SNPs for schizophrenia (14), and 3/3 SNPs for subjective well-being (15) (Figure 2). Characteristics of the genetic instruments for MR analyses were shown in Table 1. The use of pleiotropic instruments might affect the reliability of the results. Therefore, we compared the instrumental variables among the mental health disorders, and no overlap was observed. A more detailed description of these mental health disorders can be found in corresponding previous publications. The strength and bias of the mental health-related instrumental variables was evaluated by using the *F* statistics (16). For mental health disorders including in the study, range of the *F* statistics of genetic instruments was provided in Table 1, all above the recommended threshold of *F* > 10 in the MR analysis (16).

**Table 1:** Characteristics of the genetic instruments for MR analyses.

Mental health	SNPs	*F* statistics^a^	Used SNPs^b^	Participants	Ancestry	Unit^c^	Overlap^d^
Anxiety disorder	5	34 [30–44]	5/5	25,453 cases 58,113 controls	European	Odds of anxiety disorder	None/full
ADHD	12	32 [31–54]	9/8	20,183 cases 35,191 controls	Multi-ancestry (94% European)	Odds of ADHD	None/none
Autism spectrum disorder	3	33 [31–36]	2/2	18,381 cases 27,969 controls	European	Odds of autism spectrum disorder	None/none
Depression	102	38 [30–107]	100/101	246,363 cases 561,190 controls	European	Odds of depression	None/~45%
Neuroticism	84	37 [30–140]	81/84	372,903 individuals	European	1 SD increase in a weighted neuroticism sum-score	None/full
Obsessive–compulsive disorder	0	NA	NA	2,688 cases 7,037 controls	European	NA	NA
PTSD	2	33 [30–35]	2/2	23,212 cases 151,447 controls	European	Odds of PTSD	None/~30%
Schizophrenia	114	35 [28–132]	109/108	36,989 cases 113,075 controls	Multi-ancestry (97% European)	Odds of schizophrenia	None/none
Subjective well-being	3	32 [32–33]	3/3	298,420 individuals	European	1 SD increase in subjective well-being measure score	None/~10%

ADHD indicates attention deficit/hyperactivity disorder; GWAS, genome-wide association study; MR, Mendelian randomization; NA, not available; PTSD, post-traumatic stress disorder; SD, standard deviation; and SNP, single nucleotide polymorphism.

a $F=\frac{{\text{beta}}^{2}}{{\text{se}}^{2}}$, where beta is the effect size of the SNP and se its standard error on the respective mental health. *F* statistics are expressed as median [range].

b Number of SNPs used in the MR analyses (FinnGen/UK Biobank).

c Units used in the MR analyses.

d The ratio of estimated sample overlap between mental health GWAS with FinnGen/UK Biobank.

### Data sources

Summary statistics for the associations of hypertension were obtained from the FinnGen Study (fifth release) and UK Biobank. The FinnGen Study builds on samples collected by a nationwide network of Finnish biobanks, and matches the genome data with digital health care data from national health registries (17). No overlap was observed of any exposure GWAS with the FinnGen Study (Table 1). There were 42,857 patients with essential (primary) hypertension and 162,837 controls in the FinnGen Study. Essential hypertension was defined according to the following International Classification of Disease (ICD) codes: ICD-8 codes 401–404, ICD-9 codes 4,019X, 4039A, and ICD-10 code I10.

UK Biobank is a prospective cohort study which consists of more than 500,000 men and women from the UK general population aged 40 to 69 (18). There was substantial overlap between several exposure GWAS and UK Biobank (Table 1). Hypertension was defined based on discharge registries using the secondary ICD-10 code I10: essential (primary) hypertension, including 54,358 cases with essential hypertension and 408,652 controls. The data was obtained from the MR-Base platform (UKB-b:12493) (19). In addition, we used summary statistics of self-reported hypertension (199,731 cases; 343,202 controls) from UK Biobank (UKB-b:14057) as supplementary analyses. All the published GWASs had already received ethical approval from relevant institutional review boards. In the current study, we used summary-level genetic data which were publicly available. This information did not include personal, identifiable information. Thus, no additional ethics approval was required.

### Statistical analysis

For each cohort, the random-effects inverse-variance weighted (IVW) method was used to assess the associations of mental health disorders with hypertension (20). A fixed-effects meta-analysis was performed to combine the estimate from FinnGen and UK Biobank. Heterogeneity between the two cohorts was evaluated by the Cochran Q-derived *p* and *I*^2^ statistic (*p* < 0.1 or *I*^2^ > 50% as significant heterogeneity) (21).

To further validate the robustness of results, we also performed weighted median (22), MR-Egger regression (23), and MR Pleiotropy Residual Sum and Outlier (MR-PRESSO) method (24), as sensitivity analyses. The weighted median method provided consistent estimates as long as more than half instrumental variables were effective (22). MR-Egger regression explored the potential pleiotropy based on the hypotheses of independent association between genetic variants and their pleiotropic effects (23), and MR-Egger intercept test was conducted to detect the presence of directional pleiotropy (23). MR-PRESSO method was performed to detect and remove outliers, thus, correcting for horizontal pleiotropy (24).

A two-sided *p* value <0.05 was set as suggestive for significance, and we further adjusted the threshold by Bonferroni correction for number of mental health exposures (*p* < 0.05/8 = 6.25 × 10^−3^). Statistical analyses were conducted in R software (version 4.0.3; R Foundation for Statistical Computing, Vienna, Austria). MR analyses and pooled analyses in the current study were performed using the TwoSampleMR,^1^ MR-PRESSO,^2^ and metafor^3^ R packages.

## Results

**Figure 3:**
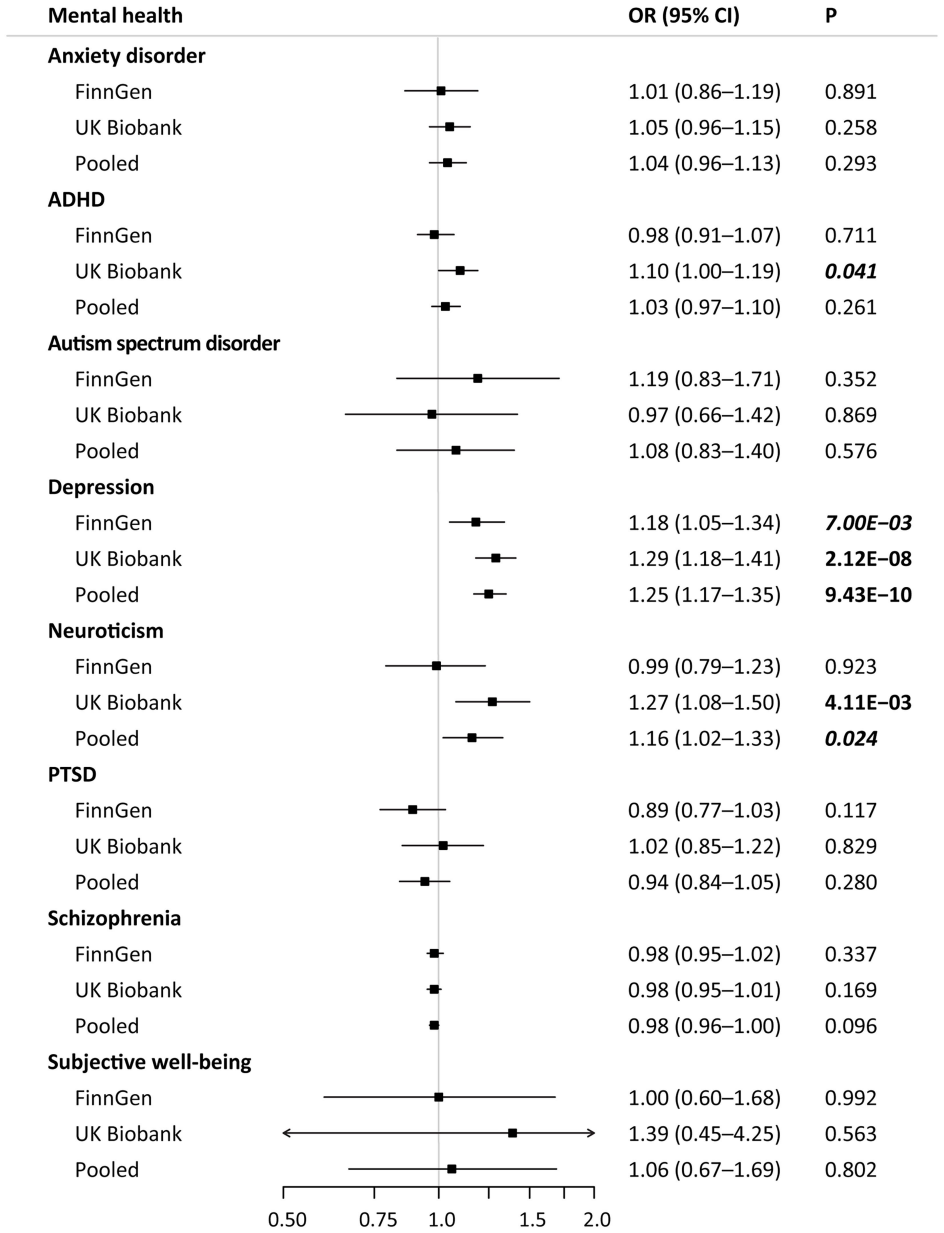
Mendelian randomization associations of mental health with hypertension in different data sources. Results are derived from the fixed-effects inverse-variance weighted analysis. ADHA indicates attention deficit/hyperactivity disorder; CI, confidence interval; OR, odds ratio; and PTSD, post-traumatic stress disorder.

The main results of MR associations of genetically predicted mental health with the risk of hypertension are shown in Figure 3. After correction for multiple testing, genetic liability to depression was significantly associated with the risk of hypertension. In the pooled analyses, genetic liability to depression was also associated with higher risk of hypertension (odds ratio [OR], 1.25; 95% confidence interval [CI], 1.17–1.35; *p* < 0.001) (Figure 3; Supplementary Figure S1). The MR analyses also showed a suggestive association that higher weighted neuroticism sum-score (1 SD increase) was associated with higher risk of hypertension (OR, 1.16; 95% CI, 1.02–1.33; *p* < 0.05), even though not reaching the Bonferroni-corrected threshold of *p* < 6.25 × 10^−3^. No significant relationships were found with genetic liability to anxiety disorder, ADHD, autism spectrum disorder, PTSD, schizophrenia, and higher subjective well-being measure score (Figure 3). Significant heterogeneity between the FinnGen and UK Biobank was observed only in the meta-analysis on ADHD (*I*^2^ = 67.3%; *p* = 0.080) and neuroticism (*I*^2^ = 68.4%; *p* = 0.075).

The main results remained stable in the weighted median and MR-PRESSO analyses. The MR-Egger intercept analyses provided no evidence of heterogeneity for the associations between mental health and hypertension (all *p* > 0.05) (Supplementary Table S1). Consistent with principal findings, the sensitivity analyses of genetically predicted mental health with self-reported hypertension in UK Biobank also presented similar results (Supplementary Table S2). Especially, considering the sample overlap (~45%, UK Biobank mainly) between the GWAS of depression and hypertension, we further conducted the supplementary analysis that including of 56 significant SNPs in 23andMe Replication and consistent results were obtained for the associations of genetically predicted depression with essential and self-reported hypertension (OR, 1.31; 95% CI, 1.13–1.51; *p* < 0.001 and OR, 1.13; 95% CI, 1.12–1.26; *p* < 0.05, respectively) (Supplementary Figure S2).

## Discussion

A comprehensive framework of MR methodologies was applied to investigate the associations of genetic liability to 8 mental health disorders with hypertension, based on genetic data from the largest published GWAS. We provided consistent evidence for the causal effect of depression on the increased risk of hypertension. The association pattern remained when it was repeated in the further supplementary analyses. Besides, a potential association of genetic liability to neuroticism with hypertension was found.

Meng et al. (2) enrolled 9 prospective studies of 22,367 normotensive participators to assess the association between depression and risk of hypertension, with a mean follow-up period of 9.6 years. Results showed that depression significantly increased risk of hypertension (OR, 1.42; 95% CI, 1.09–1.86), which was attenuated when adjusting for multiple variables. Such association was also confirmed in subsequent published work (3, 4). However, some limitations existed for the studies discussed above. First, these studies did not correct for common risk factors for hypertension fully. In addition, antidepressants were also reported to be associated with the increased risk of hypertension (4). Second, limited by the follow-up time, an inadequate duration of follow-up might underestimate the actual incidence values.

A wealth of evidence from clinical and MR study has suggested the role of depression in higher body mass index (25, 26), smoking (27, 28), excessive drinking (29, 30), physical inactivity (31), type 2 diabetes (32, 33), and lipid metabolism disorder (34). There are also studies assessing the impact of depression from a biologic mechanism perspective, finding that depression is associated with autonomic dysfunction (35), impaired endothelial function (36), platelet dysfunction (37), and elevated inflammation markers (C-reactive protein, interleukin 6, tumor necrosis factor-α, etc.) (38). In brief, the etiologic model of depression on hypertension is quite complex and cannot be explained by a single mechanism.

Our results found a suggestive association between higher weighted neuroticism sum-score and the risk of hypertension, which was consistent with reports from some longitudinal observational studies (39). However, such potential association was only observed in UK Biobank cohort. Besides, the population of neuroticism GWAS and UK Biobank was completely overlap, which may result in potential bias, and inflate the Type 1 error rate (40). Given the reasons above, it was generally insufficient to draw firm conclusion.

No evidence was found in the current study for the causal association between anxiety and hypertension. However, a recent meta-analysis that included 14 prospective studies of 686,362 participants revealed a significant anxiety-hypertension association (OR, 1.40; 95% CI, 1.23–1.59) (41). The difference between the results of this study and ours might result from the potential bias and reverse causality in observational study. Another possible reason was the low proportion of variance explained in anxiety, which would weaken the statistical power. Taking the consistent results of the vast majority of prospective studies into account (41), its potential causal effect cannot be definitively rule out.

Disagreement remained in the clinical researches regarding the effects of schizophrenia (4, 42) and subjective well-being (43–45) on hypertension. For schizophrenia, although a recent meta-analysis demonstrated no relationship with hypertension, there was a substantial heterogeneity (*I*^2^ = 90.7%) cross studies (46). Likewise, it was unclear whether subjective well-being was a protective factor of hypertension. While subjective well-being phenotype was broadly defined in the GWAS study (15), the variety of definitions among cohort studies also limited the comparison of our findings with results of these reports.

The null effects with other mental health fitted with the prospective studies, demonstrating that ADHD (47), autism spectrum disorder (3), and PTSD (3) were not associated with hypertension. It should be noted that the association of ADHD and hypertension was observed in clinical research but did not reach statistical significance after adjusting for body mass index (47). We also acknowledge that the analyses of ADHD, autism spectrum disorder, PTSD and subjective well-being suffered from insufficient statistical power and hence might report false-negative associations. Further GWAS with larger sample were needed to identify more significant loci.

The major strength of the current study was the design of MR study, which strengthened the causal inference to estimate the non-biased causal effect compared with observational studies. Besides, most studies included in the current analysis were with large sample sizes, which may guarantee the reliability of results. In addition, combining sensitivity analyses based on multiple statistical models with further supplementary analyses from different datasets, we provided more solid and reliable genetic evidence for the causal association between mental health and hypertension. Meanwhile, several limitations should be acknowledged. First, there was substantial overlap between several exposure GWAS and hypertension in UK Biobank. However, the association pattern of depression with hypertension remained when using variants significant in 23andMe replication. In addition, robust instruments and large-scale consortia also decreased the bias and the chance of Type 1 error to a certain degree (40). Second, the power for certain analysis, like the analysis for anxiety might be inadequate given a small number of used instrumental variables which explains a limited phenotypic variance. Besides, the strength of association was not very strong, especially for neuroticism. Third, the lack of individual-level genotyping data made it impossible to assess the association of mental health and hypertension across age groups and genders. Fourth, the population of GWAS used in this study was mainly of European descent, which reduced the population stratification bias, but at the same time limited the generalizability of the results to other populations.

## Conclusion

In summary, the current study provides consistent evidence for the causal effect of genetic liability to depression on hypertension, which shows the clinical significance regarding blood pressure measurement and monitoring in patients with depression.

## Data availability statement

The datasets presented in this study can be found in online repositories. The names of the repository/repositories and accession number(s) can be found in the article/Supplementary material.

## Ethics statement

Ethical review and approval was not required for the study on human participants in accordance with the local legislation and institutional requirements. Written informed consent for participation was not required for this study in accordance with the national legislation and the institutional requirements.

## Author contributions

NH, YL, FY, and HC contributed to the conception or design of the work. FY, YL, and HM contributed to the acquisition, analysis, or interpretation of data for the work. NH, YL, and FY wrote the manuscript. HM, ZH, NH, and HC revised the manuscript and gave critical suggestions. All gave final approval and agree to be accountable for all aspects of work ensuring integrity and accuracy.

## Funding

This work was supported by grants from Key Laboratory of Precision Medicine for Atherosclerotic Diseases of Zhejiang Province, China (Grant No. 2022E10026), Major Project of Science and Technology Innovation 2025 in Ningbo, China (Grant No. 2021Z134), Public Science and Technology Projects of Ningbo (Grant No. 202002 N3175) and Key research and development project of Zhejiang Province, China (Grant No. 2021C03096).

## Conflict of interest

The authors declare that the research was conducted in the absence of any commercial or financial relationships that could be construed as a potential conflict of interest.

## Publisher’s note

All claims expressed in this article are solely those of the authors and do not necessarily represent those of their affiliated organizations, or those of the publisher, the editors and the reviewers. Any product that may be evaluated in this article, or claim that may be made by its manufacturer, is not guaranteed or endorsed by the publisher.

## Footnotes

^1^ https://github.com/MRCIEU/TwoSampleMR

^2^ https://github.com/rondolab/MR-PRESSO

^3^ https://github.com/wviechtb/metafor
