# Supplementary material for: Genetic liability to mental disorders in relation to the risk of hypertension
Source: Front Cardiovasc Med. 2023 Feb 27;10:1087251. doi: 10.3389/fcvm.2023.1087251 (PMC10008891; doi:10.3389/fcvm.2023.1087251)
Supplement: Supplementary file 10 [file Data_Sheet_1.PDF]

Supplemental Materials

Table S1. Sensitivity analyses for the Mendelian randomization associations of mental health with risk of hypertension

		Sensitivity analyses									
		IVW		Weighted median		MR-Egger			MR-PRESSO		
**Mental health**	SNPs	OR (95% CI)	P	OR (95% CI)	P	OR (95% CI)	P	P of MR-Egger intercept	SNPs	OR (95% CI)	P
Anxiety disorder											
FinnGen	5	1.01 (0.86 – 1.19)	0.891	0.97 (0.87 – 1.08)	0.599	0.56 (0.06 – 5.34)	0.647	0.640	4*	0.93 (0.86 – 1.01)	0.177
UK Biobank	5	1.05 (0.96 – 1.15)	0.258	1.00 (0.92 – 1.08)	0.960	1.45 (0.41 – 5.11)	0.607	0.656	5	1.05 (0.96 – 1.15)	0.258
ADHD											
FinnGen	9	0.98 (0.91 – 1.07)	0.711	1.06 (0.94 – 1.19)	0.321	1.25 (0.89 – 1.75)	0.240	0.199	9	0.98 (0.91 – 1.07)	0.711
UK Biobank	8	1.10 (1.00 – 1.19)	***0.041***	1.11 (1.02 – 1.21)	***0.012***	0.98 (0.67 – 1.43)	0.907	0.568	8	1.10 (1.00 – 1.19)	***0.041***
Autism spectrum											
FinnGen	2	1.19 (0.83 – 1.71)	0.352	NA	NA	NA	NA	NA	NA	NA	NA
UK Biobank	2	0.97 (0.66 – 1.42)	0.869	NA	NA	NA	NA	NA	NA	NA	NA
Depression											
FinnGen	100	1.18 (1.05 – 1.34)	***7.00E-03***	1.21 (1.06 – 1.38)	**5.92E-03**	0.69 (0.33 – 1.46)	0.338	0.157	97*	1.24 (1.11 – 1.39)	**2.68E-04**
UK Biobank	101	1.29 (1.18 – 1.41)	**2.12E-08**	1.33 (1.21 – 1.46)	**1.85E-09**	0.97 (0.58 – 1.64)	0.919	0.285	97*	1.33 (1.23 – 1.44)	**3.32E-10**
Neuroticism											
FinnGen	81	0.99 (0.79 – 1.23)	0.923	1.01 (0.79 – 1.28)	0.959	1.77 (0.51 – 6.14)	0.368	0.352	79*	0.99 (0.80 – 1.22)	0.906
UK Biobank	84	1.27 (1.08 – 1.50)	**4.11E-03**	1.29 (1.10 – 1.51)	**1.98E-03**	0.99 (0.42 – 2.37)	0.986	0.572	78*	1.34 (1.17 – 1.54)	**8.16E-05**
PTSD											
FinnGen	2	0.89 (0.77 – 1.03)	0.117	NA	NA	NA	NA	NA	NA	NA	NA
UK Biobank	2	1.02 (0.85 – 1.22)	0.829	NA	NA	NA	NA	NA	NA	NA	NA
Schizophrenia											
FinnGen	109	0.98 (0.95–1.02)	0.337	0.99 (0.94–1.03)	0.508	0.96 (0.84–1.10)	0.538	0.694	109	0.98 (0.95–1.02)	0.337

UK Biobank	108	0.98 (0.95–1.01)	0.169	1.01 (0.98–1.04)	0.454	1.01 (0.89–1.15)	0.824	0.584	105*	0.98 (0.96–1.01)	0.257
Subjective well-being											
FinnGen	3	1.00 (0.60 – 1.68)	0.992	NA	NA	NA	NA	NA	NA	NA	NA
UK Biobank	3	1.39 (0.45 – 4.25)	0.563	NA	NA	NA	NA	NA	NA	NA	NA

*One or more outliers excluded.

P values below the Bonferroni-corrected threshold for number of mental health of 6.25×10^{-3} are shown in bold and suggestive *P* values between 0.05 and 6.25×10^{-3} are shown in bold-italic.

ADHA indicates attention deficit/hyperactivity disorder; CI, confidence interval; IVW, inverse-variance weighted; MR, Mendelian randomization; MR-PRESSO, MR-pleiotropy residual sum and outlier; NA, not available; OR, odds ratio; PTSD, post-traumatic stress disorder; and SNPs, single nucleotide polymorphisms.

Table S2. Sensitivity analyses for the Mendelian randomization associations of mental health with risk of self-reported hypertension in UK Biobank

	UK Biobank Self-reported hypertension (cases: 199,731, controls: 343,202)		
**Mental health**	SNPs	OR (95% CI)	P
Anxiety disorder	5	1.02 (0.96–1.08)	0.543
ADHD	8	1.01 (0.96–1.06)	0.746
Autism spectrum disorder	2	0.98 (0.70–1.39)	0.924
Depression	101	1.12 (1.05–1.19)	**2.76×10^{-4}**
Neuroticism	84	1.11 (1.03–1.20)	**4.11×10^{-3}**
PTSD	2	0.97 (0.90–1.05)	0.460
Schizophrenia	108	0.98 (0.96–1.00)	0.078
Subjective well-being	3	1.61 (0.87–2.98)	0.127

Results are derived from the fixed-effects inverse-variance weighted analysis. *P* values below the Bonferroni-corrected threshold for number of mental health of 6.25×10^{-3} are shown in bold and suggestive *P* values between 0.05 and 6.25×10^{-3} are shown in bold-italic. ADHA indicates attention deficit/hyperactivity disorder; CI, confidence interval; OR, odds ratio; PTSD, post-traumatic stress disorder; and SNPs, single nucleotide polymorphisms.

Table S3. Instrumental variables of mental health disorders in the Mendelian randomization analyses.

Phenotype	SNP	EA	OA	beta	se	pval
depression	rs301799	T	C	-0.025	0.004	1.36E-12
depression	rs1002656	T	C	-0.027	0.004	3.74E-12
depression	rs1466887	T	C	-0.020	0.004	4.12E-08
depression	rs11579246	A	G	0.038	0.006	5.71E-10
depression	rs1890946	T	C	-0.024	0.004	2.68E-11
depression	rs10789214	T	C	0.019	0.004	4.44E-08
depression	rs2568958	A	G	0.037	0.004	8.47E-25
depression	rs10890020	A	G	-0.028	0.004	4.03E-15
depression	rs113188507	A	G	0.022	0.004	1.87E-08
depression	rs10913112	T	C	-0.026	0.004	3.40E-13
depression	rs72710803	A	C	-0.041	0.006	5.29E-11
depression	rs169235	A	G	-0.023	0.004	2.98E-08
depression	rs17641524	T	C	-0.032	0.004	1.52E-13
depression	rs12052908	A	T	-0.022	0.004	4.44E-10
depression	rs1568452	T	C	0.025	0.004	8.12E-12
depression	rs7585722	T	C	-0.027	0.005	2.68E-08
depression	rs1226412	T	C	0.026	0.004	3.46E-09
depression	rs62188629	A	G	0.024	0.004	7.13E-10
depression	rs4346585	T	C	-0.024	0.004	7.13E-10
depression	rs13084037	A	G	-0.025	0.004	7.08E-09
depression	rs7624336	T	G	0.024	0.004	3.96E-08
depression	rs141954845	A	G	0.023	0.004	8.15E-10
depression	rs6783233	T	C	0.022	0.004	2.90E-08
depression	rs1095626	T	C	-0.026	0.004	7.13E-14
depression	rs7685686	A	G	0.020	0.004	2.57E-08
depression	rs34937911	T	C	0.030	0.006	4.13E-08
depression	rs45510091	A	G	0.045	0.008	1.83E-08
depression	rs35553410	T	C	-0.024	0.004	1.42E-09
depression	rs7659414	A	C	-0.020	0.004	1.20E-08
depression	rs60157091	T	C	0.020	0.004	1.42E-08
depression	rs3099439	T	C	-0.028	0.004	5.05E-15
depression	rs10061069	C	G	-0.028	0.004	8.15E-11
depression	rs30266	A	G	0.031	0.004	1.45E-16
depression	rs11135349	A	C	-0.030	0.004	6.04E-17
depression	rs200949	A	G	0.048	0.005	2.53E-19
depression	rs9363467	T	C	0.024	0.004	6.44E-11
depression	rs7758630	A	T	-0.023	0.004	5.56E-10
depression	rs1933802	C	G	-0.022	0.004	2.57E-10
depression	rs2876520	C	G	-0.023	0.004	2.29E-10

depression	rs725616	T	C	0.020	0.004	1.87E-08
depression	rs2029865	A	T	-0.020	0.004	1.20E-08
depression	rs3823624	T	C	0.027	0.005	1.99E-09
depression	rs2043539	A	G	0.027	0.004	9.89E-15
depression	rs2247523	C	G	-0.021	0.004	4.38E-09
depression	rs16887442	T	C	0.020	0.004	8.62E-09
depression	rs58104186	A	G	0.024	0.004	1.82E-11
depression	rs7807677	T	C	0.024	0.004	1.82E-11
depression	rs7837935	T	G	-0.029	0.005	3.34E-09
depression	rs67436663	C	G	-0.026	0.004	9.37E-10
depression	rs1354115	A	C	0.021	0.004	7.08E-09
depression	rs1982277	T	C	0.028	0.004	1.45E-11
depression	rs263645	A	T	0.022	0.004	3.70E-10
depression	rs3793577	A	G	-0.023	0.004	8.41E-11
depression	rs59283172	A	G	-0.033	0.006	1.02E-08
depression	rs34653192	C	G	-0.023	0.004	2.23E-09
depression	rs7030813	T	C	0.025	0.004	3.07E-12
depression	rs10817969	T	G	0.026	0.004	3.11E-11
depression	rs913930	A	G	-0.021	0.004	2.42E-08
depression	rs2670139	T	C	-0.027	0.004	1.21E-10
depression	rs997934	T	C	0.020	0.004	4.81E-08
depression	rs1021363	A	G	0.030	0.004	4.41E-16
depression	rs1448938	A	G	0.021	0.004	1.30E-09
depression	rs2509805	T	C	0.022	0.004	9.17E-09
depression	rs198457	T	C	-0.029	0.005	2.99E-10
depression	rs58621819	A	T	-0.025	0.004	1.57E-08
depression	rs7117514	A	G	-0.020	0.004	7.29E-09
depression	rs7932640	T	C	0.028	0.004	1.62E-15
depression	rs61902811	A	G	-0.026	0.004	1.40E-12
depression	rs2187490	T	G	-0.034	0.006	3.82E-08
depression	rs57344483	A	G	-0.038	0.007	1.82E-08
depression	rs78337797	T	G	0.031	0.006	3.37E-08
depression	rs56314503	T	G	-0.025	0.004	2.95E-10
depression	rs10774600	T	C	-0.027	0.005	3.39E-08
depression	rs3213572	A	G	0.022	0.004	7.61E-10
depression	rs1409379	T	C	0.025	0.004	1.67E-09
depression	rs1343605	A	C	0.031	0.004	6.23E-18
depression	rs9592461	A	G	0.022	0.004	9.10E-10
depression	rs9545360	A	C	-0.027	0.005	5.02E-09
depression	rs4772087	T	C	0.023	0.004	3.91E-10
depression	rs61990288	A	G	-0.026	0.004	1.68E-13
depression	rs1956373	T	G	-0.023	0.004	2.06E-08
depression	rs1152578	T	C	-0.022	0.004	6.36E-10

depression	rs1045430	T	G	-0.025	0.004	7.31E-13
depression	rs10149470	A	G	-0.027	0.004	3.72E-14
depression	rs8037355	T	C	-0.023	0.004	3.94E-11
depression	rs34488670	T	C	-0.025	0.004	6.03E-09
depression	rs7193263	A	G	-0.024	0.004	4.33E-10
depression	rs7198928	T	C	0.024	0.004	4.45E-11
depression	rs7200826	T	C	0.028	0.004	3.74E-12
depression	rs56887639	A	G	-0.028	0.004	1.51E-12
depression	rs12923444	A	C	-0.021	0.004	1.30E-09
depression	rs75581564	A	G	0.030	0.005	3.17E-08
depression	rs12967855	A	G	0.027	0.004	1.18E-12
depression	rs7227069	A	G	0.024	0.004	1.50E-11
depression	rs62091461	T	C	-0.025	0.004	1.95E-09
depression	rs12966052	C	G	-0.031	0.005	1.25E-11
depression	rs12967143	C	G	-0.031	0.004	3.70E-16
depression	rs7241572	A	G	0.028	0.004	2.70E-10
depression	rs33431	T	C	0.020	0.004	4.81E-08
depression	rs143186028	T	G	0.028	0.005	2.29E-09
depression	rs12624433	A	G	0.023	0.004	7.44E-09
depression	rs5995992	T	C	-0.027	0.004	1.30E-11
anxiety	rs4855559	T	G	-0.120	0.022	3.70E-08
anxiety	rs2861139	C	T	0.130	0.022	2.60E-09
anxiety	rs3807866	A	G	0.120	0.022	4.80E-08
anxiety	rs10959883	C	T	-0.150	0.023	2.90E-11
anxiety	rs1187280	G	A	0.130	0.022	6.60E-09
ADHD	rs11420276	G	GT	0.107	0.015	2.14E-13
ADHD	rs1222063	A	G	0.096	0.017	3.07E-08
ADHD	rs9677504	A	G	0.117	0.021	1.39E-08
ADHD	rs4858241	T	G	0.079	0.014	1.74E-08
ADHD	rs28411770	T	C	0.086	0.015	1.15E-08
ADHD	rs4916723	A	C	-0.077	0.014	1.58E-08
ADHD	rs5886709	G	GTC	0.076	0.013	1.66E-08
ADHD	rs74760947	A	G	-0.180	0.032	1.35E-08
ADHD	rs11591402	A	T	-0.093	0.016	1.34E-08
ADHD	rs1427829	A	G	0.080	0.013	1.82E-09
ADHD	rs281324	T	C	-0.074	0.013	2.68E-08
ADHD	rs212178	A	G	-0.115	0.020	7.68E-09
ASD	rs10099100	C	G	0.084	0.015	1.07E-08
ASD	rs910805	A	G	-0.096	0.016	2.04E-09
ASD	rs71190156	GTTTTTTT	G	-0.078	0.014	2.75E-08
neuroticism	rs12137398	T	C	0.017	0.003	3.36E-09
neuroticism	rs11576073	A	G	0.015	0.002	2.95E-11
neuroticism	rs17096778	G	A	-0.033	0.006	3.99E-09

neuroticism	rs7536102	C	T	-0.015	0.003	5.51E-09
neuroticism	rs2488401	T	C	-0.018	0.003	9.69E-10
neuroticism	rs17432675	C	T	-0.014	0.003	1.73E-08
neuroticism	rs4396680	A	G	0.016	0.003	7.42E-09
neuroticism	rs10188070	A	G	-0.014	0.002	2.55E-09
neuroticism	rs2042555	A	G	0.017	0.002	2.84E-13
neuroticism	rs1226574	C	G	-0.018	0.003	2.44E-10
neuroticism	rs10497655	C	T	-0.014	0.002	4.08E-09
neuroticism	rs78720888	A	C	-0.018	0.003	3.06E-08
neuroticism	rs6737187	A	G	-0.014	0.003	1.44E-08
neuroticism	rs9867227	A	G	0.017	0.003	4.90E-11
neuroticism	rs1542212	G	T	0.016	0.002	8.62E-12
neuroticism	rs836927	A	C	0.016	0.002	2.54E-12
neuroticism	rs59382200	G	A	-0.013	0.002	4.70E-08
neuroticism	rs655836	C	T	-0.016	0.002	1.16E-12
neuroticism	rs4585149	T	C	-0.017	0.003	8.27E-09
neuroticism	rs6828271	C	T	-0.014	0.002	1.29E-09
neuroticism	rs1501673	A	G	0.020	0.003	1.98E-09
neuroticism	rs11240962	A	C	0.017	0.003	4.66E-10
neuroticism	rs4868748	G	A	0.015	0.003	3.30E-09
neuroticism	rs2031595	T	C	-0.015	0.003	5.41E-09
neuroticism	rs2071303	C	T	-0.014	0.002	9.73E-09
neuroticism	rs200965	A	G	-0.017	0.003	9.42E-09
neuroticism	rs28986304	C	T	0.021	0.004	9.30E-10
neuroticism	rs2503775	A	G	0.021	0.003	4.53E-10
neuroticism	rs240769	A	C	-0.014	0.002	1.37E-09
neuroticism	rs2056477	C	G	-0.015	0.003	2.83E-08
neuroticism	rs11509880	A	G	0.015	0.002	8.32E-10
neuroticism	rs10274968	C	T	-0.013	0.002	2.25E-08
neuroticism	rs274632	A	C	-0.013	0.002	1.97E-08
neuroticism	rs35048193	T	G	0.016	0.002	4.83E-13
neuroticism	rs2407746	G	C	0.017	0.003	3.93E-12
neuroticism	rs7818437	C	T	0.019	0.003	3.43E-10
neuroticism	rs28639817	G	A	0.020	0.003	3.14E-09
neuroticism	rs2380937	C	T	-0.014	0.002	4.80E-09
neuroticism	rs62550480	T	C	-0.018	0.003	1.70E-10
neuroticism	rs10811883	C	T	0.017	0.002	1.64E-12
neuroticism	rs10119773	A	G	-0.016	0.002	2.89E-11
neuroticism	rs12344656	A	G	-0.014	0.002	6.39E-09
neuroticism	rs78046549	T	C	0.024	0.004	1.55E-10
neuroticism	rs75614054	T	C	0.030	0.004	3.69E-14
neuroticism	rs7025144	T	C	0.018	0.003	5.88E-12
neuroticism	rs860626	G	T	-0.015	0.003	4.95E-09

neuroticism	rs4757136	T	A	0.016	0.002	4.78E-12
neuroticism	rs297343	T	G	0.016	0.002	3.75E-11
neuroticism	rs1806153	T	G	0.017	0.003	3.87E-10
neuroticism	rs12283653	G	A	0.013	0.002	3.21E-08
neuroticism	rs7107356	A	G	-0.016	0.002	6.39E-12
neuroticism	rs10896636	G	C	0.016	0.002	2.50E-11
neuroticism	rs10830220	G	C	0.014	0.002	1.77E-09
neuroticism	rs7111031	C	A	-0.019	0.002	1.99E-16
neuroticism	rs11605020	G	A	-0.013	0.002	2.26E-08
neuroticism	rs167915	T	A	-0.013	0.002	3.62E-08
neuroticism	rs9971907	G	A	0.013	0.002	2.82E-08
neuroticism	rs11608355	C	T	0.019	0.002	2.83E-15
neuroticism	rs3741475	A	G	0.017	0.003	1.03E-09
neuroticism	rs6490177	A	C	0.018	0.003	6.10E-10
neuroticism	rs3124426	C	T	0.015	0.003	1.89E-08
neuroticism	rs4444227	T	C	-0.016	0.003	1.95E-09
neuroticism	rs1892350	G	A	0.014	0.002	1.03E-09
neuroticism	rs7999314	T	G	0.014	0.002	1.40E-08
neuroticism	rs1778377	A	T	-0.015	0.003	1.72E-09
neuroticism	rs11627348	A	C	0.019	0.003	4.43E-09
neuroticism	rs1275411	C	T	-0.014	0.002	8.06E-09
neuroticism	rs9671386	G	A	-0.019	0.003	8.46E-13
neuroticism	rs12442330	T	C	0.013	0.002	2.74E-08
neuroticism	rs4360891	C	T	0.014	0.002	1.26E-09
neuroticism	rs3785232	C	T	-0.018	0.003	1.29E-12
neuroticism	rs3751855	C	T	-0.015	0.002	2.25E-10
neuroticism	rs2042395	G	A	0.015	0.003	3.11E-08
neuroticism	rs12938775	G	A	0.015	0.002	2.20E-11
neuroticism	rs35982947	C	A	-0.014	0.002	6.02E-09
neuroticism	rs77804065	T	C	0.032	0.003	3.85E-31
neuroticism	rs56084168	T	C	-0.024	0.003	7.43E-14
neuroticism	rs10460051	T	C	0.014	0.002	2.69E-10
neuroticism	rs11082011	C	T	0.022	0.002	2.58E-20
neuroticism	rs11875397	A	T	-0.017	0.003	7.82E-09
neuroticism	rs4632195	C	T	-0.014	0.002	8.69E-10
neuroticism	rs12958048	A	G	0.015	0.002	8.98E-10
neuroticism	rs2425752	T	C	0.017	0.003	7.90E-11
neuroticism	rs9611519	T	C	0.018	0.003	1.25E-12
PTSD	rs34517852	A	T	0.109	0.019	3.16E-09
PTSD	rs9364611	T	C	-0.124	0.023	4.36E-08
schizophrenia	rs4648845	T	C	0.070	0.012	8.70E-10
schizophrenia	rs1498232	T	C	0.067	0.011	2.86E-09
schizophrenia	rs11210892	A	G	-0.068	0.011	3.39E-10

schizophrenia	rs12129573	A	C	0.075	0.011	2.03E-12
schizophrenia	rs76869799	C	G	-0.167	0.030	2.64E-08
schizophrenia	rs1702294	T	C	-0.120	0.014	3.36E-19
schizophrenia	rs140505938	T	C	-0.090	0.014	4.49E-10
schizophrenia	rs6670165	T	C	0.072	0.013	4.45E-08
schizophrenia	rs7523273	A	G	0.061	0.011	4.47E-08
schizophrenia	rs10803138	A	G	-0.069	0.012	2.03E-08
schizophrenia	rs77149735	A	G	0.275	0.047	3.73E-09
schizophrenia	rs14403	T	C	-0.068	0.012	4.42E-08
schizophrenia	rs11682175	T	C	-0.069	0.010	1.47E-11
schizophrenia	rs75575209	A	T	-0.103	0.019	3.95E-08
schizophrenia	rs3768644	A	G	-0.101	0.017	7.39E-09
schizophrenia	rs2909457	A	G	-0.058	0.011	4.62E-08
schizophrenia	rs11693094	T	C	-0.074	0.010	1.53E-12
schizophrenia	rs59979824	A	C	-0.065	0.011	8.41E-09
schizophrenia	rs6434928	A	G	-0.074	0.011	2.06E-11
schizophrenia	rs6704641	A	G	0.078	0.014	8.33E-09
schizophrenia	rs11685299	A	C	-0.063	0.011	1.12E-08
schizophrenia	rs6704768	A	G	-0.073	0.010	2.32E-12
schizophrenia	rs17194490	T	G	0.096	0.014	2.69E-11
schizophrenia	rs4330281	T	C	-0.062	0.011	4.64E-09
schizophrenia	rs75968099	T	C	0.082	0.011	1.05E-13
schizophrenia	rs2535627	T	C	0.069	0.010	4.26E-11
schizophrenia	rs832187	T	C	-0.061	0.011	1.43E-08
schizophrenia	rs7432375	A	G	-0.069	0.011	7.26E-11
schizophrenia	rs9841616	A	T	-0.078	0.014	2.35E-08
schizophrenia	rs215411	A	T	0.062	0.011	3.06E-08
schizophrenia	rs35518360	A	T	-0.154	0.020	7.98E-15
schizophrenia	rs10520163	T	C	0.063	0.010	1.47E-09
schizophrenia	rs1106568	A	G	-0.068	0.012	9.47E-09
schizophrenia	rs1501357	T	C	-0.077	0.013	5.05E-09
schizophrenia	rs4391122	A	G	-0.081	0.010	1.10E-14
schizophrenia	rs16867576	A	G	0.096	0.017	4.61E-09
schizophrenia	rs4388249	T	C	0.073	0.013	3.05E-08
schizophrenia	rs10043984	T	C	0.067	0.012	1.09E-08
schizophrenia	rs3849046	T	C	0.061	0.010	4.67E-09
schizophrenia	rs79212538	T	G	0.144	0.025	7.00E-09
schizophrenia	rs111294930	A	G	0.090	0.014	1.06E-10
schizophrenia	rs2973155	T	C	-0.069	0.011	1.11E-10
schizophrenia	rs12522290	C	G	0.081	0.014	1.99E-08
schizophrenia	rs11740474	A	T	-0.060	0.011	3.15E-08
schizophrenia	rs115329265	A	G	0.186	0.016	3.48E-31
schizophrenia	rs1339227	T	C	-0.060	0.011	2.69E-08

schizophrenia	rs117074560	T	C	-0.164	0.027	1.64E-09
schizophrenia	rs12704290	A	G	-0.101	0.016	3.33E-10
schizophrenia	rs6466055	A	C	0.066	0.011	1.13E-09
schizophrenia	rs211829	T	C	0.059	0.010	3.71E-08
schizophrenia	rs13240464	T	C	0.080	0.011	3.03E-13
schizophrenia	rs7801375	A	G	-0.079	0.015	4.42E-08
schizophrenia	rs3735025	T	C	0.064	0.011	3.28E-09
schizophrenia	rs10503253	A	C	0.070	0.013	1.06E-08
schizophrenia	rs73229090	A	C	-0.097	0.017	2.10E-08
schizophrenia	rs6984242	A	G	-0.061	0.011	5.97E-09
schizophrenia	rs7819570	T	G	0.076	0.014	1.22E-08
schizophrenia	rs36068923	A	G	-0.084	0.013	2.61E-11
schizophrenia	rs4129585	A	C	0.083	0.010	1.74E-15
schizophrenia	rs11139497	A	T	0.067	0.011	3.61E-09
schizophrenia	rs7893279	T	G	0.118	0.017	1.97E-12
schizophrenia	rs7907645	T	G	0.134	0.020	1.27E-11
schizophrenia	rs11191419	A	T	-0.099	0.011	6.20E-19
schizophrenia	rs55833108	T	G	0.072	0.013	2.23E-08
schizophrenia	rs11027857	A	G	0.062	0.010	2.55E-09
schizophrenia	rs9420	A	G	0.066	0.011	2.24E-09
schizophrenia	rs12421382	T	C	-0.061	0.011	3.70E-08
schizophrenia	rs2514218	T	C	-0.076	0.011	2.75E-11
schizophrenia	rs77502336	C	G	0.064	0.011	7.54E-09
schizophrenia	rs55661361	A	G	-0.077	0.011	2.80E-12
schizophrenia	rs10791097	T	G	0.073	0.010	1.09E-12
schizophrenia	rs75059851	A	G	0.087	0.013	3.87E-11
schizophrenia	rs2007044	A	G	-0.092	0.011	3.22E-18
schizophrenia	rs2239063	A	C	0.065	0.011	1.93E-08
schizophrenia	rs679087	A	C	-0.061	0.011	3.91E-08
schizophrenia	rs324017	A	C	-0.064	0.011	2.13E-08
schizophrenia	rs12826178	T	G	-0.167	0.024	2.02E-12
schizophrenia	rs4240748	C	G	-0.059	0.011	4.59E-08
schizophrenia	rs10860964	T	C	0.058	0.011	4.84E-08
schizophrenia	rs4766428	T	C	0.066	0.011	1.40E-09
schizophrenia	rs2851447	C	G	-0.089	0.012	1.86E-14
schizophrenia	rs2068012	T	C	-0.069	0.012	1.41E-08
schizophrenia	rs2332700	C	G	0.070	0.012	4.86E-09
schizophrenia	rs2693698	A	G	-0.063	0.011	4.80E-09
schizophrenia	rs12887734	T	G	0.084	0.012	1.36E-13
schizophrenia	rs56205728	A	G	0.071	0.012	4.18E-09
schizophrenia	rs12903146	A	G	0.065	0.010	3.38E-10
schizophrenia	rs12148337	T	C	0.058	0.010	1.79E-08
schizophrenia	rs8042374	A	G	0.089	0.012	2.44E-13

schizophrenia	rs190065944	A	G	0.075	0.014	4.71E-08
schizophrenia	rs950169	T	C	-0.080	0.012	1.62E-11
schizophrenia	rs4702	A	G	-0.081	0.011	8.30E-14
schizophrenia	rs9922678	A	G	0.065	0.011	1.28E-08
schizophrenia	rs7405404	T	C	0.074	0.012	1.01E-09
schizophrenia	rs12691307	A	G	0.070	0.011	4.55E-11
schizophrenia	rs12325245	A	T	-0.083	0.015	1.87E-08
schizophrenia	rs8044995	A	G	0.078	0.014	1.51E-08
schizophrenia	rs4523957	T	G	0.069	0.011	2.86E-10
schizophrenia	rs8082590	A	G	-0.063	0.011	1.77E-08
schizophrenia	rs78322266	T	G	0.172	0.030	1.32E-08
schizophrenia	rs9636107	A	G	-0.073	0.010	3.34E-12
schizophrenia	rs72934570	T	C	-0.136	0.021	1.97E-11
schizophrenia	rs715170	T	C	-0.067	0.012	1.27E-08
schizophrenia	rs2905426	T	G	-0.068	0.011	3.63E-10
schizophrenia	rs2053079	A	G	-0.071	0.012	4.49E-09
schizophrenia	rs56873913	T	G	0.069	0.013	4.69E-08
schizophrenia	rs6065094	A	G	-0.075	0.011	1.46E-11
schizophrenia	rs7267348	T	C	-0.065	0.012	4.56E-08
schizophrenia	rs9607782	A	T	0.083	0.012	2.07E-11
schizophrenia	rs1023500	T	C	0.073	0.013	3.43E-08
schizophrenia	rs6002655	T	C	0.064	0.010	1.71E-09
schizophrenia	rs12845396	A	T	-0.054	0.010	2.21E-08
schizophrenia	rs1378559	T	C	0.086	0.012	1.61E-12
schizophrenia	rs5937157	T	G	-0.064	0.010	1.98E-10
SWB	rs3756290	A	G	-0.018	0.003	9.55E-09
SWB	rs2075677	A	G	0.018	0.003	1.49E-08
SWB	rs4958581	T	C	0.015	0.003	2.29E-08

SNP, single nucleotide polymorphism; EA, effect allele; OA, other allele; se, standard error; pval, *p*-value.

Figure S1. Scatter plots of SNP effects regarding the associations of depression with hypertension in (A) FinnGen and (B) UK Biobank, with the slope of line corresponding to estimated Mendelian randomization effect derived from inverse-variance-weighted analyses

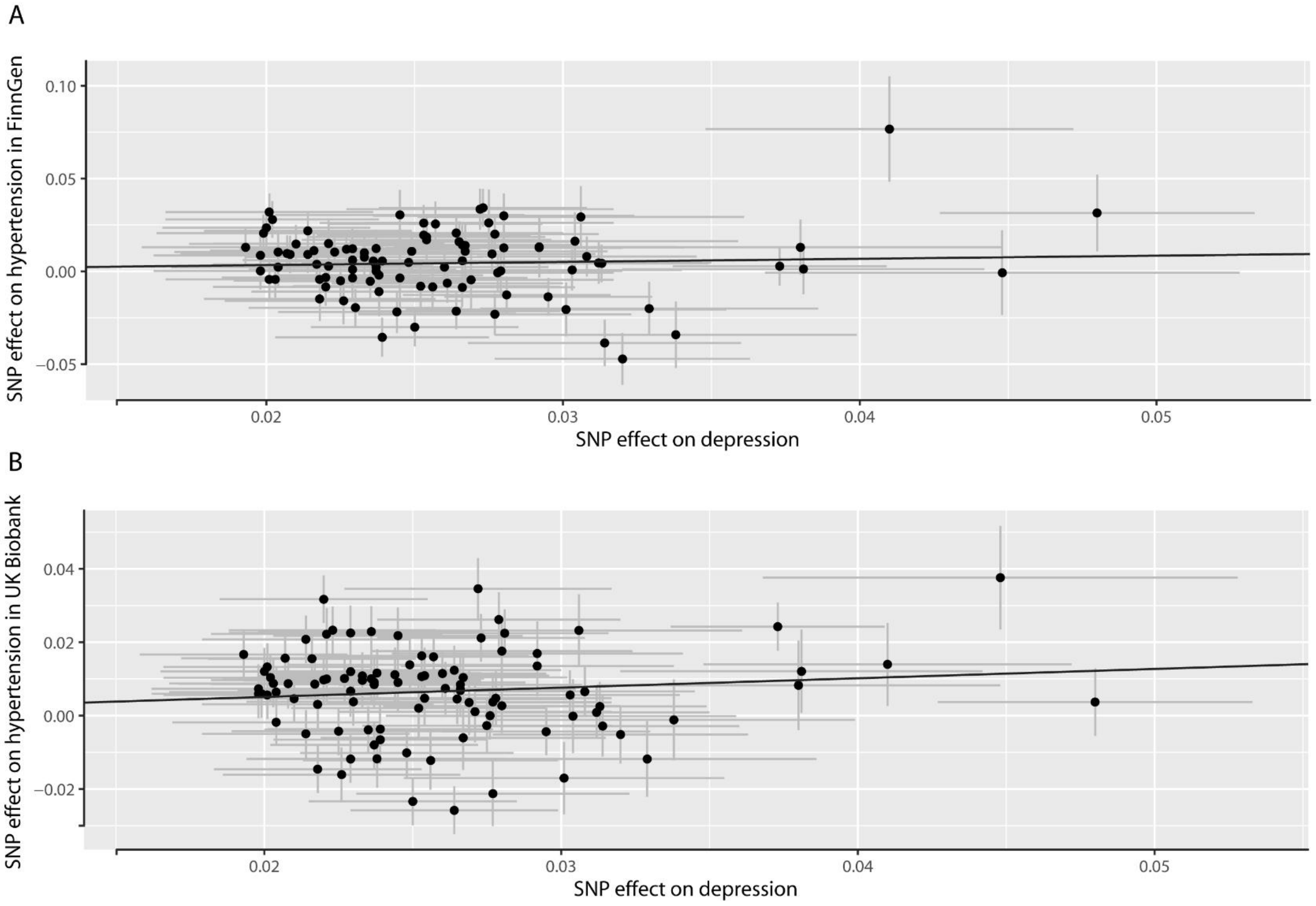

Figure S2. Mendelian randomization association of depression with hypertension

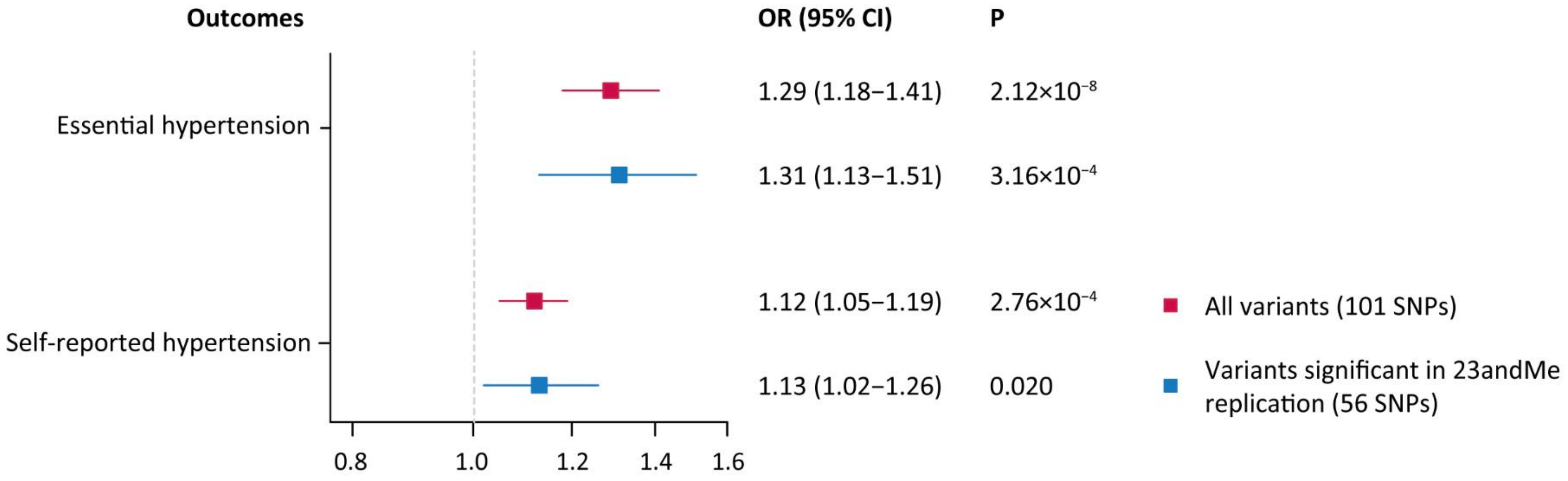

CI indicates confidence interval; OR, odds ratio; and SNPs, single nucleotide polymorphisms.
